# Integrated Analysis of Tumor Mutation Burden and Immune Infiltrates in Hepatocellular Carcinoma

**DOI:** 10.3390/diagnostics12081918

**Published:** 2022-08-08

**Authors:** Yulan Zhao, Ting Huang, Pintong Huang

**Affiliations:** Department of Ultrasound in Medicine, Second Affiliated Hospital of Zhejiang University School of Medicine, Hangzhou 310000, China

**Keywords:** hepatocellular carcinoma, tumor mutation burden, immune cells, The Cancer Genome Atlas, CIBERSORT

## Abstract

Tumor mutation burdens (TMBs) act as an indicator of immunotherapeutic responsiveness in various tumors. However, the relationship between TMBs and immune cell infiltrates in hepatocellular carcinoma (HCC) is still obscure. The present study aimed to explore the potential diagnostic markers of TMBs for HCC and analyze the role of immune cell infiltration in this pathology. We used OA datasets from The Cancer Genome Atlas database. First, the “maftools” package was used to screen the highest mutation frequency in all samples. R software was used to identify differentially expressed genes (DEGs) according to mutation frequency and perform functional correlation analysis. Then, the gene ontology (GO) enrichment analysis was performed with “clusterProfiler”, “enrichplot”, and “ggplot2” packages. Finally, the correlations between diagnostic markers and infiltrating immune cells were analyzed, and CIBERSORT was used to evaluate the infiltration of immune cells in HCC tissues. As a result, we identified a total of 359 DEGs in this study. These DEGs may affect HCC prognosis by regulating fatty acid metabolism, hypoxia, and the P53 pathway. The top 15 genes were selected as the hub genes through PPI network analysis. *SRSF1*, *SNRPA1*, and *SRSF3* showed strong similarities in biological effects, NCBP2 was demonstrated as a diagnostic marker of HCC, and high NCBP2 expression was significantly correlated with poor over survival (OS) in HCC. In addition, NCBP2 expression was correlated with the infiltration of B cells (r = 0.364, *p* = 3.30 × 10^−12^), CD8^+^ T cells (r = 0.295, *p* = 2.71 × 10^−8^), CD4^+^ T cells, (r = 0.484, *p* = 1.37 × 10^−21^), macrophages (r = 0.551, *p* = 1.97 × 10^−28^), neutrophils (r = 0.457, *p* = 3.26 × 10^−19^), and dendritic cells (r = 0.453, *p* = 1.97 × 10^−18^). Immune cell infiltration analysis revealed that the degree of central memory T-cell (Tcm) infiltration may be correlated with the HCC process. In conclusion, NCBP2 can be used as diagnostic markers of HCC, and immune cell infiltration plays an important role in the occurrence and progression of HCC.

## 1. Introduction

Hepatocellular carcinoma (HCC) is one of the most common and aggressive malignancies in the digestive system and contributes to a severe global disease burden worldwide [1]. It ranked sixth in global incidence (4.7%) and was the third leading cause of cancer-related deaths (8.3%) in 2020, according to a recent study [2]. The prognosis of patients is usually driven by the tumor stage. The 5-year survival rates for local disease exceed 70%; however, the median survival time of advanced-stage HCC patients is only 1 year [3]. Although the survival situation has improved, benefiting from advancements in medical treatments [4], approximately 2/3 of HCC patients are diagnosed at advanced stages, and the median overall survival rate remains at a low level [5]. Therefore, there is an urgent need to explore the potential molecular mechanisms of tumor progression to develop better therapeutic strategies and investigate the potential benefits of adjuvant systemic therapies.

The molecular mechanisms contributing to the development of HCC are extremely complex and involve various genetic abnormalities, such as the dysregulation of signaling pathways, genomic instability, single-nucleotide polymorphisms (SNPs), and somatic mutations [6,7]. The somatic mutations were reported frequently among HCC patients, and the landscape was complicated, including somatic mutations that occur in multitudes of genes accompanied by the changes of multiple signaling pathways [8], which contribute to various molecular heterogeneities that remain poorly understood. With the rise of high-throughput sequencing technology, a large number of databases based on TCGA (The Cancer Genome Atlas) and GEO (Gene Expression Omnibus) datasets have emerged, making it convenient for us to investigate the complex relationships between HCC and the underlying oncogenic somatic mutation molecular mechanisms. Our results may provide new insight into novel diagnostic and prognostic values for HCC.

In addition, recent studies have demonstrated that TMB(Tumor mutation burden) was correlated with immune cell infiltration and subtypes [9,10]. TMB is defined as the frequency of gene mutations (total count of variants/the whole length of exons), including translocation, deletion, and insertion mutations, in addition to other mutations that appear in the somatic-gene-coding region, with an average 1 Mb-base range for the tumor genome, and it is used as a biomarker to predict the sensitivity, efficacy, and treatment outcomes of immune checkpoint inhibitors (ICPIs) [11,12]. The tumor cell carries new antigens generated by somatic mutations on the cell surface that may be recognized by the immune system, further making the tumor cell a target for activated immune cells [13]. To date, there have been numerous studies focusing on the relationship between TMB and immunotherapy in diverse cancers [14,15,16], and accumulating evidence indicates that a high tumor mutation burden confers an increased immune reaction to tumors and a better response to ICPI treatment [17]. However, the prognostic value of TMB in HCC has not yet been clearly determined.

In the present study, we downloaded The Cancer Genome Atlas HCC data sets using R software package and other online databases to investigate the association of genes bearing important mutations contributing to TMBs with clinical and genomic features in HCC patients. We performed gene ontology (GO) term enrichment and protein–protein interaction (PPI) analysis and constructed functional networks related to NCBP2 in HCC. Finally, the relationship between NCBP2 and immune cell infiltration in the HCC was also analyzed. The findings from the present study suggest that NCBP2 influences the prognosis of HCC patients via its interaction with infiltrating immune cells.

## 2. Materials and Methods

### 2.1. Data Download

The Cancer Genome Atlas (TCGA, https://cancergenome.nih.gov/) (accessed on 1 March 2021) database provides publicly available cancer genome datasets. TCGA database contains 369 cases of LICH tissue samples. We used R language RTCGToolbox package from TCGA database (https://portal.gdc.cancer.gov/) (accessed on 1 March 2021) to download Liver Cancer (LIHC) gene expression spectrum and clinical data as the training sets. We included a total of 364 cases of LIHC samples in the present study. We used the maftools package to screen the 20 genes with the highest mutation frequencies in all samples, and we visualized the mutation situations and frequencies of all samples. We grouped all samples according to the genes with the highest mutation frequencies.

### 2.2. Data Preprocessing and Differentially Expressed Gene (DEG) Screening

We used affy package (R version 3.6.3; TUNA Team, Tsinghua University, Beijing, China) to perform background correction and data normalization, and we screened differentially expressed genes (DEGs) by using limma software package. The screening criteria were: | log2 fold change (log2FC) | > 1, adjust *p* < 0.05. We used univariate Cox regression to screen out prognostic Genes. We used the intersection Search Tool (http://string-db.org; Version: 11.0) (accessed on 1 March 2021) for the Retrieval of Separated Genes (STRING) to predict the protein–protein interaction (PPI) network. We used Cytoscape to visualize complex networks and integrate them with data of any attribute type. Gene ontology (GO) is a common method used to annotate genes and their products. This method is often used to annotate large-scale genes, determining molecular function (MF) and biological process (BP). We used cellular components (CCs) for a GO analysis of intersecting genes.

### 2.3. GSEA and GSVA Analysis

We performed GSEA and GSVA analysis to explore the important pathway of enrichment between the two groups. The reference gene set was H.all.v.7.1.symbols.gmt. We replaced 1000 genomes to achieve standardized enrichment scores for each analysis. We considered a nominal *p* < 0.05 and a false discovery rate < 0.05 as significant results. We used clusterProfiler and GSVA packages for GSVA analysis, and we considered adj.*p* value < 0.05 as a meaningful pathway.

### 2.4. Verification of Differential Expression of NCBP2

We used GEPIA2 (http://gepia2.cancer-pku.cn/) (accessed on 1 March 2021) to verify the differential expression between liver cancer and other cancer and paracancer samples in the database. We applied the box plot module of the GEPIA2 database to explore the expression level of NCBP2 in various cancer datasets, including the GTEx and TCGA databases, and we also analyzed the expression levels of NCBP2 in different stages of liver cancer through a Stage Plot module. Then, we used the Survival Map module to investigate the overall survival (OS) rates in liver and other cancers. Significance level is 0.05.

### 2.5. Prognostic Analysis

The Kaplan–Meier mapping platform is able to assess the effects of more than 50,000 genes on survival in 21 cancer types. The primary purpose of this tool is the discovery and validation of survival biomarkers based on meta-analysis. We explored the correlation between NCBP2 and prognosis of liver cancer in Kaplan–Meier mapping platform to verify the relationship between NCBP2 and liver cancer prognosis.

### 2.6. Expression Verification of NCBP2 in Cells and Tissues

The Human Protein Atlas is an open-access database used to map all human proteins in organ tissues and cells, and integrates various omics techniques. We detected the mRNA expression of NCBP2 in organ tissues and large tumors using the Human Protein Atlas and TIMER database. We used this database to preliminarily verify the expression levels of NCBP2 in cells and tissues.

### 2.7. Correlation Analysis between NCBP2 and Immunity

We applied “corrplot package” to further investigate the infiltration conditions of immune cells and the relationship between NCBP2 and immune cells in liver cancer. We constructed a correlation heatmap to visualize the correlation of 22 types of infiltrating immune cells in liver cancer. Then, we performed Spearman correlation analyses using “ggstatsplot” package (https://github.com/IndrajeetPatil/ggstatsplot) (accessed on 1 March 2021) to investigate the relationship between the levels of NCBP2 and immune cells.

## 3. Results

### 3.1. Landscape of Gene Mutation Files in LIHC

**Figure 1:**
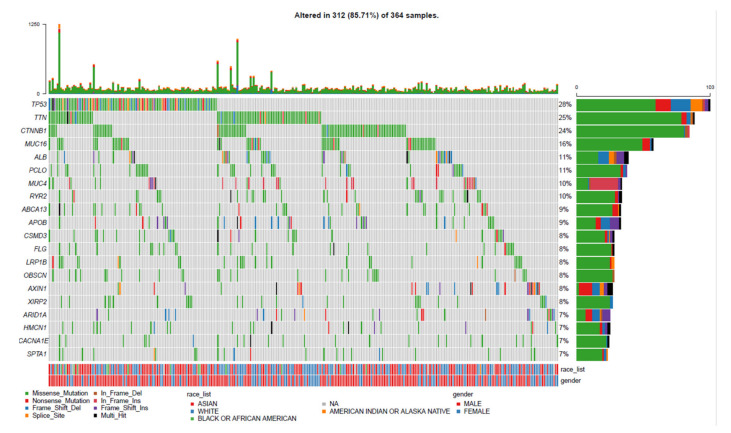
Landscape profile of top 20 mutated genes in 364 LIHC from TCGA database. Mutations of each gene in each sample are shown in waterfall plot. Each column presents specific sample, each line presents mutated gene, and name is listed on left. Different forms of somatic mutations and percentages of gene mutation types are shown on right (color version of figure is available online). LIHC: Liver Cancer; TCGA: The Cancer Genome Atlas.

To investigate the mutation profile among the TCGA-LIHC cohort, we used the RTCGToolbox package of R language to acquire the LIHC gene expression spectrum and clinical data as the training set from TCGA database (https://portal.gdc.cancer.gov/) (accessed on 1 March 2021). The maftools package was used to screen the top 20 genes with high mutation frequencies in all samples, and waterfall plots were utilized to visualize the mutation landscapes of the genes. The results of the somatic mutation profiles in 364 cases of LIHC samples included in the present study showed that around 312 (85.71%) samples possessed somatic mutations. As for the top 20 mutated genes shown in Figure 1, we discovered that gene *TP53* mutated most frequently, approximately accounting for 28% of mutations, followed by *TTN* (25%), *CTNNB1* (24%), *MUC16* (16%), *ALB* (11%), *PCLO* (11%), *MUC4* (10%), *RYR2* (10%), *ABCA13* (9%) and *APOB* (9%), *CSMD3* (8%), *FLG* (8%), *LRP1B* (8%), *OBSCN* (8%), *AXIN1* (8%), *XIRP2* (8%), *ARID1A* (7%), *HMCN1* (7%), *CACNA1E* (7%), and *SPTA1* (7%). Missense mutations were the most frequent among these alterations.

### 3.2. Data Preprocessing and Screening of DEGs

**Figure 2:**
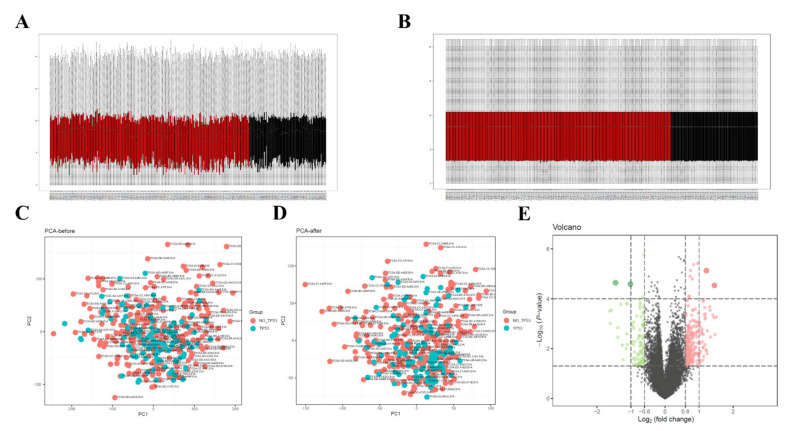
Data preprocessing and differential expression analysis. (**A**,**B**) Box chart of gene expression among high- and low-TMB groups. Black dots represent mean values of gene expression after sample normalization before (**A**) and after (**B**) sample normalization. (**C**,**D**) before (**C**) and after (**D**) principal component analyses (PCA) of gene expression between high- and low-TMB groups. (**E**) Volcano map of DEGs; red represents upregulated differential genes, green represents downregulated differential genes, and grey represents no-significant-difference genes. TMB: Tumor Mutation Burden.

All samples obtained from above were divided into high- and low-TMB groups according to the median TMB threshold, and we further evaluated the missing data and normalization for data preprocessing. The box chart results showed that similar levels of data points were achieved after correcting the mean value of the gene expression, and the data homogenization was credible (Figure 2A,B). The gene expression matrix was then merged for further normalization. The PCA results indicated that the clustering of samples was more obvious between the two groups after homogenization (Figure 2C,D), and the results suggested that the sample data source included in the present study was reliable and could be used for further analysis. After data preprocessing, we identified 2171 DEGs between high- and low-TMB groups with |Log FC| > 1 and *p* value < 0.05 through the limma package of R software. The result was presented via a volcano map (Figure 2E), in which green dots represent downregulated genes, red dots represent upregulated genes, and black dots represent unchanged genes.

### 3.3. Joint Screening of Genes, PPI Network Construction, Hub Genes Screening, and Similarities

**Figure 3:**
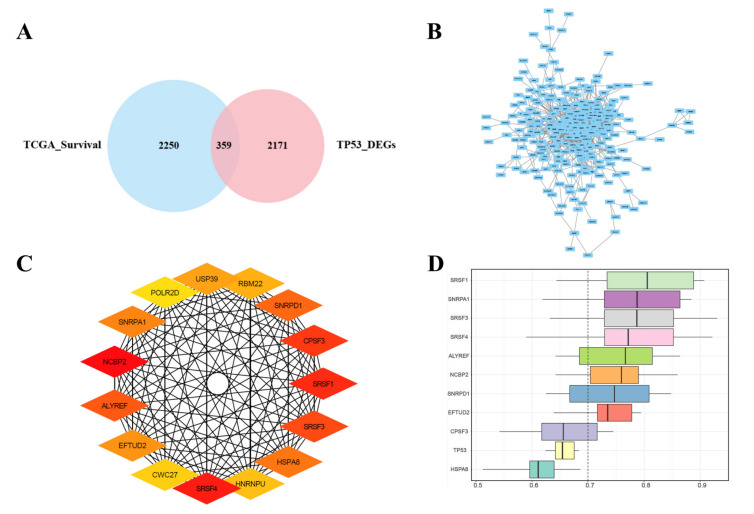
Joint screening of DEGs, Protein–protein interaction (PPI), hub DEGs, and functional similarity analysis of DEGs. (**A**) Venn diagram of DEGs between high- and low-TMB groups and the prognosis-related genes with *p* value less than 0.05 in Cox univariate analysis obtained from TCGA. Middle part represents overlap of two groups of data. (**B**) Gene interaction network of 359 prognosis-related DEGs visualized with PPI network. (**C**) Interaction network of 15 DEGs scored by maximum correlation coefficient; the darker the color, the higher the MCC algorithm score. (**D**) Functional similarities of 11 hub genes—dashed line represents cut-off value of similarity. DEGs: Differentially Expressed Genes; TCGA: The Cancer Genome Atlas; MCC: Matthews correlation coefficient.

In order to explore more accurate genes related to the prognosis of patients with HCC, intersection analysis was conducted on the identified differentially expressed genes between the high- and low-TMB groups, and the prognosis-related genes with *p* values < 0.05 in univariate Cox analysis were obtained from TCGA database. The combined results revealed that a total of 359 differentially expressed genes were identified following the intersection of 2171 DEGs between high- and low-TMB groups, with 2250 genes related to prognosis and survival (Figure 3A). Search Tool for the Retrieval of Interacting Genes (STRING) (http://string db.org; Version: 11.0) (accessed on 1 March 2021) is an online tool for predicting protein–protein interaction (PPI) networks. An analysis of functional interactions between proteins can provide more information into the mechanisms of disease occurrence or development. Through Cytoscape and its plug-in cytoHubba, we constructed the PPI network of DEGs related to prognosis obtained above (Figure 3B). The top 15 genes were selected as the hub genes through the MCC cytoHubba plugin with the highest correlation scores in this PPI network: *USP39*, *RBM22*, *SNRPD1*, *CPSF3*, *SRSF1*, *SRSF3*, *HSPA8*, *HNRNPU*, *SRSF4*, *CWC27*, *EFTUD2*, *ALYREF*, *NCBP2*, *SNRPA1*, and *POLR2D* (Figure 3C). To further explore the closeness of the correlation between hub DEGs, which were ranked on the basis of average functional similarity, the results suggested that *SRSF1*, *SNRPA1*, *SRSF3*, *SRSF4*, *ALYREF*, *NCBP2*, *SNRPD1*, and *EFTUD2* were found to be hub genes with cut-off values greater than 0.7, and *SRSF1*, *SNRPA1*, and *SRSF3* showed a strong similarity in biological effects (Figure 3D).

### 3.4. Functional Correlation Analysis

**Figure 4:**
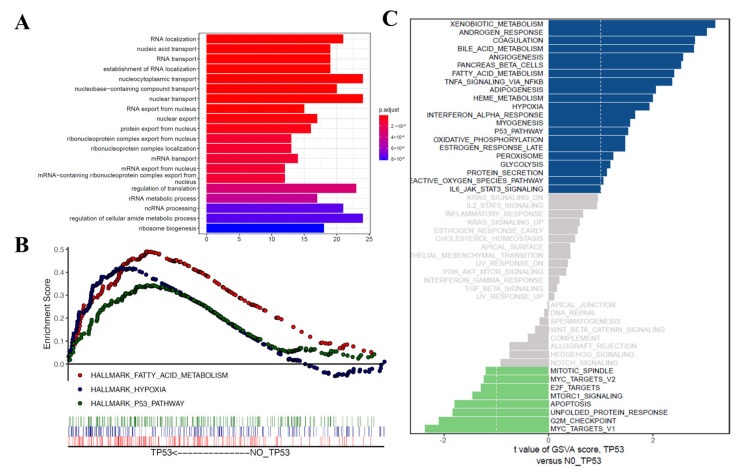
GO, GSEA, and GSVA analyses. (**A**) Significantly enriched gene ontology terms in categories BP. (**B**) GSEA analysis based on h.all.v7.1.symbols.gmt. (**C**) GSVA analysis based on h.all.v7.1.symbols.gmt. GO: Gene ontology; GSEA: gene set enrichment analysis; GSVA: gene set variation analysis.

A total of 359 differentially expressed genes related to prognosis in HCC samples were further subjected to GO analysis. The results suggested that in the biological process (BP) category, these prognosis-related differentially expressed genes were mainly correlated with RNA localization and the transport and export of components in the nucleus (Figure 4A). In order to explore the important pathway of enrichment between the two groups, the gene set enrichment analysis (GSEA) of gene expression profiles was used to identify differentially enriched signaling pathways between patients in high- and low-TMB groups. The results suggested that the enriched functions and pathways in the high-TMB group mainly involved fatty acid metabolism, hypoxia, and the P53 pathway (Figure 4B). The results of gene set variation analysis (GSVA) revealed that androgen response, coagulation, bile acid metabolism, angiogenesis, pancreas beta cells, fatty acid metabolism, TNFA signaling via NFKB and adipogenesis were enriched in the high-TMB group (Figure 4C).

### 3.5. The mRNA Expression Level of NCBP2 in Hepatocellular Carcinoma

**Figure 5:**
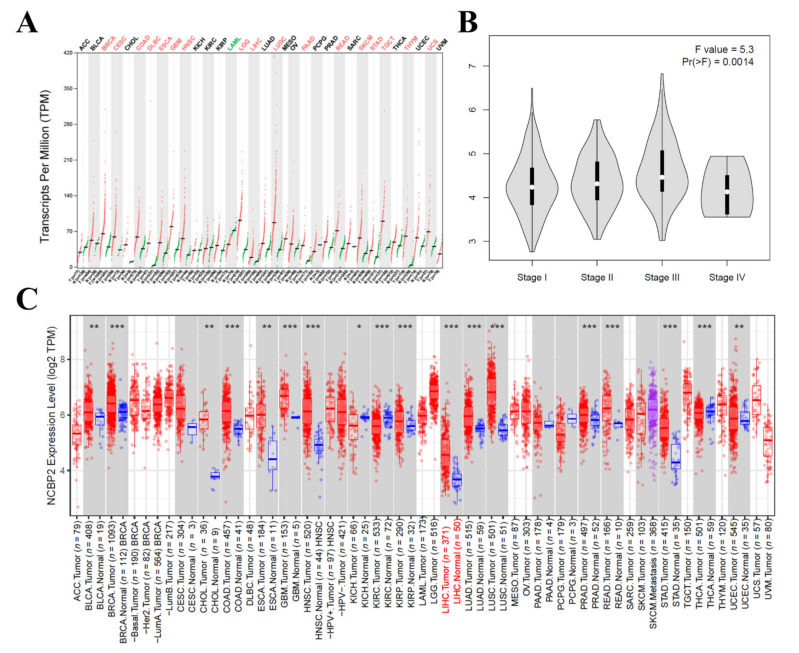
NCBP2 expression levels in HCC. (**A**) Expression patterns of NCBP2 in 33 cancer types and paired non-tumor samples. (**B**) Violin plots reveal relationship between NCBP2 expression and LIHC staging. (**C**) Human NCBP2 expression levels in different tumor types determined by TIMER (* *p* < 0.05, ** *p* < 0.01, *** *p* < 0.001). TIMER: Tumor Immune Estimation Resource.

To further explore the mRNA expression level of NCBP2 in hepatocellular carcinoma, we performed a verification to investigate the differential mRNA expression between HCC tumor samples and adjacent normal samples in the GEPIA2 (http://gepia2.cancer-pku.cn/) (accessed on 1 March 2021) and TIMER databases (https://cistrome.shinyapps.io/timer/) (accessed on 1 March 2021). As a result, the GEPIA-based analysis indicated that NCBP2 was upregulated in 17 of 33 cancer types, including hepatocellular carcinoma, which was computed in the form of transcripts per million compared with adjacent tissues (Figure 5A). In addition, the mRNA expression of NCBP2 was significantly different among different stages of HCC (F value = 0.53, Pr(>F) = 0.0014) (Figure 5B). Finally, we evaluated the NCBP2 mRNA expression using the RNA-seq data in TIMER database. The result also indicated that the mRNA expression of NCBP2 was overexpressed in hepatocellular carcinoma tissues compared with adjacent tissues, and NCBP2 mRNA expression was also overexpressed in other cancer types, such as BLCA (bladder urothelial carcinoma), BRCA (breast invasive carcinoma), CHOL (cholangiocarcinoma), COAD (colon adenocarcinoma), ESCA (esophageal carcinoma), GBM (glioblastoma multiforme), HNSC (head and neck squamous cell carcinoma), KIRP (kidney renal papillary cell carcinoma), LUAD (lung adenocarcinoma), LUSC (lung squamous cell carcinoma), PRAD (prostate adenocarcinoma), READ (rectum adenocarcinoma), STAD (stomach adenocarcinoma), and UCEC (uterine corpus endometrial carcinoma), but downregulated in KICH (kidney chromophobe) and KIRC (kidney renal clear cell carcinoma) (Figure 5C). In summary, all these results indicate that the mRNA expression level of NCBP2 is significantly overexpressed in HCC.

### 3.6. Correlations between the mRNA Expression Level of NCBP2 and Survival in HCC Patients

**Figure 6:**
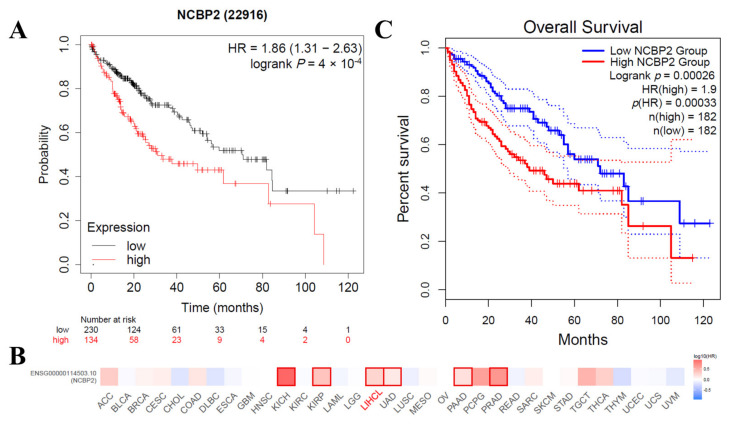
Kaplan–Meier survival curves comparing high and low expressions of NCBP2 in different databases. (**A**) Kaplan–Meier survival curves of LIHC in PrognoScan. (**B**) Relationship between NCBP2 expression and survival prognosis of each cancer in TCGA. (**C**) Kaplan–Meier survival curves of LIHC in Kaplan–Meier Plotter. Number at risk represent number of people exposed to outcome risk at each time point.

To further investigate the relationship of the mRNA expression level of NCBP2 with the survival situation in HCC patients, the Kaplan–Meier Plotter, which is based on the transcriptome data mainly extracted from GEO, EGA, and TCGA, was used to assess the NCBP2-related survival rate. As a result, we firstly identified NCBP2 as a detrimental prognostic factor in LIHC (Overall Survival (OS): HR = 1.86, 95% CI from 1.31 to 2.63, log-rank *p* = 4 × 10^4^) (Figure 6A). Then, we further investigated the prognostic value of NCBP2 expression for pan-cancer in another database. The correlation between NCBP2 expression and the prognosis of each cancer were investigated, and the result suggested that NCBP2 expression was significantly related to a total of six cancer types, including KICH, KIRP, LICH, LUAD, PAAD, and PRAD (Figure 6B), and the expression level of NCBP2 was negatively correlated with over survival. Among those cancers, NCBP2 played a detrimental role in LIHC according to the GEPIA2 database (OS: total number = 364, HR = 1.9, log-rank *p* = 0.00026) (Figure 6C). In summary, we identified NCBP2 as a detrimental biomarker for the survival prognosis of HCC.

### 3.7. Protein Expression Level of NCBP2 in Human Tissue and Cell Lines

**Figure 7:**
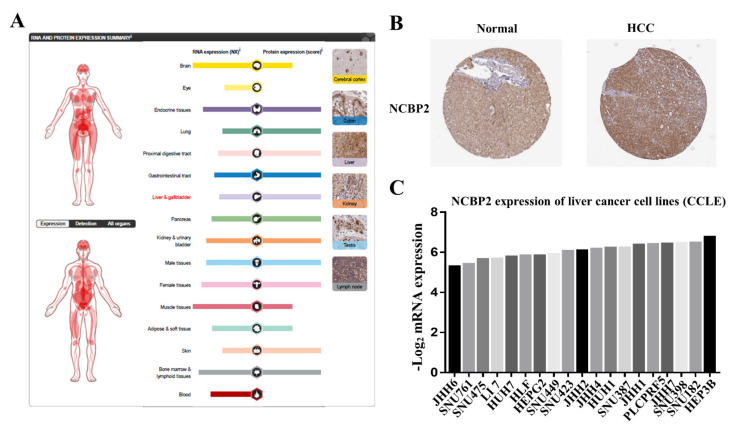
NCBP2 protein expression in human tissues and cell lines. (**A**) NCBP2 protein expression in normal human tissues based on The Human Protein Atlas (THPA). (**B**) NCBP2 expression assessed using immunohistochemistry in normal and liver cancer tissues. (**C**) NCBP2 gene expression profiles of 19 liver cancer cell lines based on Cancer Cell Line Encyclopedia (CCLE) database.

After investigating the mRNA expression pattern of NCBP2 in various databases, we further explored the protein expression pattern of NCBU2 in cell lines and human tissue in The Human Protein Atlas database (THPA), including tumor samples and normal adjacent specimens. The results confirmed that the protein level of NCBP2 was expressed moderately less in normal liver tissues compared with other normal tissues (Figure 7A), and the immunohistochemical analysis demonstrated that NCBP2 was overexpressed in HCC tissue relative to the normal adjacent sample (Figure 7B). The expression level of NCBP2 in liver cancer cell lines was analyzed using the CCLE online platform, and the result showed that the liver cancer cell lines with the highest expression of NCBP2 was from the HEP3B cell, and the lowest was from the JHH6 cell (Figure 7C).

### 3.8. Relationship between the NCBP2 Expression and TP53 Mutation with Immune Makers

**Figure 8:**
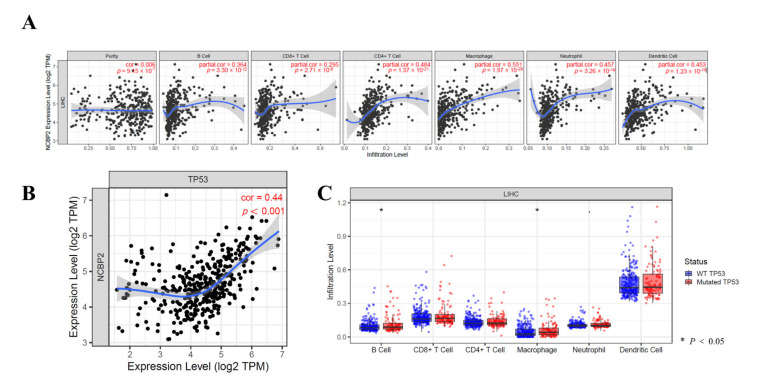
Correlation of NCBP2 expression and TP53 mutation with immune infiltration levels in LIHC. (**A**) Relationship of NCBP2 expression with immune infiltration. (**B**) Relationship of NCBP2 expression with TP53. (**C**) Correlation between TP53 mutation and immune infiltration.

**Figure 9:**
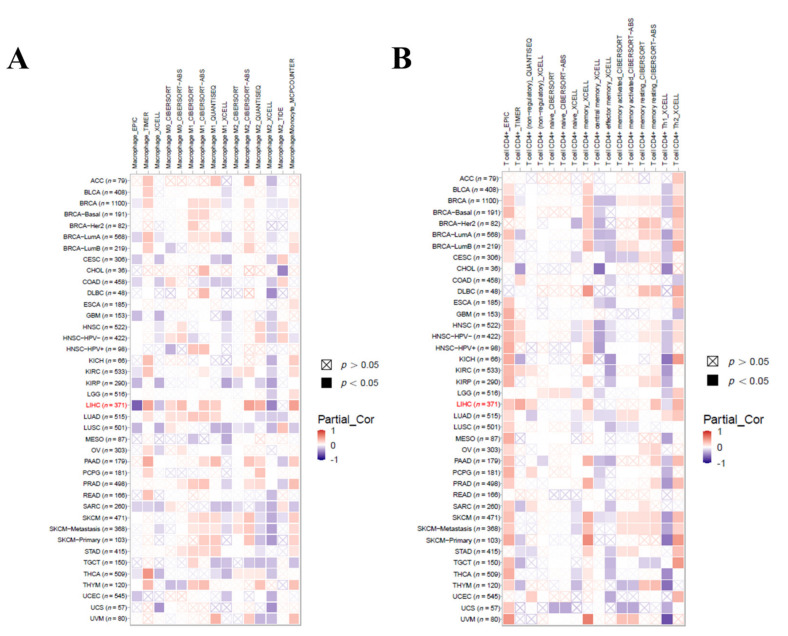
Relationship of NCBP2 expression with immune infiltration level in diverse cancer types (TIMER 2.0). (**A**) Macrophage immune infiltration level. (**B**) CD4^+^ T-cell immune infiltration level.

Immune infiltration was involved with hepatocellular carcinoma progression. Since NCBP2 expression was related to the prognostic of hepatocellular carcinoma, the relationship between 22 infiltrating immune cells and the NCBP2 expression was investigated by the TIMER database. The results suggested that, after adjustments for tumor purity, the NCBP2 expression was positively associated with all immune cells, including B cells (r = 0.364, *p* = 3.30 × 10^−12^), CD8^+^ T cells (r = 0.295, *p* = 2.71 × 10^−8^), CD4^+^ T cells, (r = 0.484, *p* = 1.37 × 10^−21^), macrophages (r = 0.551, *p* = 1.97 × 10^−28^), neutrophils (r = 0.457, *p* = 3.26 × 10^−19^), and dendritic cells (r = 0.453, *p* = 1.97 × 10^−18^) (Figure 8A). Intriguingly, we also found that the expression of NCBP2 was positively associated with TP53 (Figure 8B). After the prognosis of hepatocellular carcinoma related to the genetic mutations, among which TP53 represented a primary concern, we further investigated the relationship between the TP53 mutation and immune infiltration. The results showed that B cells and macrophages were significantly higher in the TP53 mutant than the wildtype; however, the rest of the immune cells, including CD8^+^ T cells, CD4^+^ T cells, neutrophils, and dendritic cells, were not statistically significant with TP53 (Figure 8C). We further analyzed the relationship between NCBP2 expression with macrophages and CD4^+^ T cell infiltration levels in diverse cancer types using the TIMER 2.0 database. The results indicated that NCBP2 expression was positively correlated with the immune infiltration levels of macrophages (Figure 9A) and CD4^+^ T cells (Figure 9B) across most tumor types, with the highest correlation shown in LIHC. Univariate and multivariate COX regression also showed that the stage of HCC, CD8^+^ T cells, and the expression of NCBP2 were the independent indicators for predicting the prognosis of OS patients (Table 1).

### 3.9. Immune Cell Infiltration Analysis in LIHC

**Figure 10:**
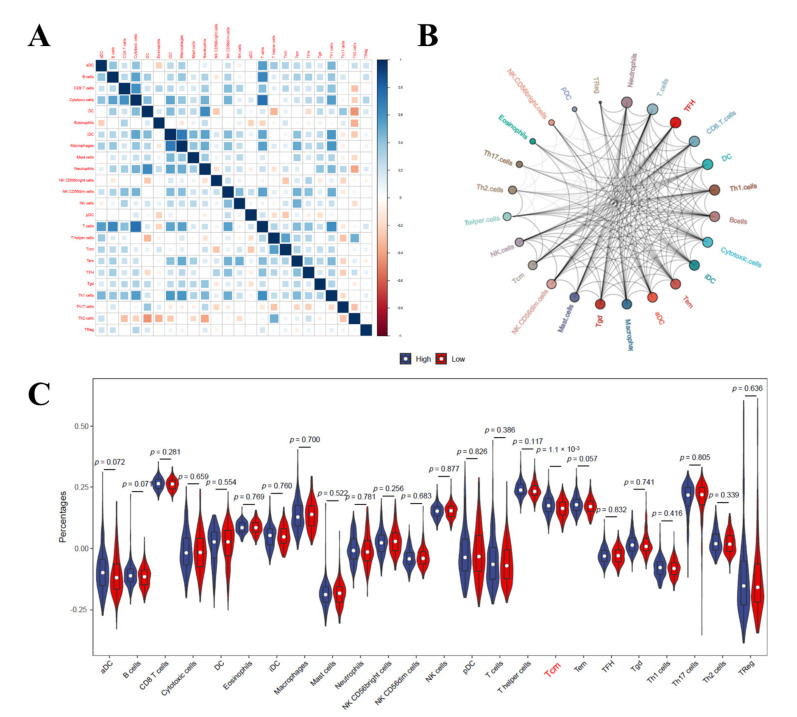
Correlation plots of immune cell infiltration analysis in LIHC. (**A**) Correlation heat map of 22 immune cells. Blue indicates positive correlation, red indicates negative correlation. Size of colored squares indicates strength of correlation. (**B**) Network diagram of 24 immune cell types. The circle size indicates the strength of interaction. (**C**) Violin diagram shows the difference of 24 types of immune cell infiltration in high mutation frequency of TP53 versus low mutation frequency of TP53.

Finally, we evaluated the infiltration of immune cells in LIHC. The results of the correlation heatmap between the 22 types of immune cells revealed that T cells had a significant positive correlation with cytotoxic cells and type 1 T-helper cells (Th1), and the macrophages and immature dendritic cells (iDC) also had a positive correlation. Type 2 T-helper cells (Th2) had a significant negative correlation with dendritic cells (DCs) and neutrophils, and the T-helper cells also had a negative correlation with DCs (Figure 10A). The immune cell interaction network results suggested that neutrophils, T cells, and follicular helper T cells (TFH) have strong relationships with other immune cells, but that regulatory cells (TReg) and plasmacytoid dendritic cells (pDC) have a weak relationship with other immune cells (Figure 10B). The violin plot of the immune cell infiltration results revealed that the degree of central memory T-cell (Tcm) infiltration was higher than in the low mutation frequencies of the TP53 samples (*p* < 0.05) (Figure 10C).

## 4. Discussion

HCC is one of the most common malignant tumors. According to Global Cancer Statistics 2020, there were 906,000 new cases of HCC worldwide each year, causing about 830,000 deaths [2]. The main risk factors for HCC are chronic infection with the hepatitis B (HBV) or C virus (HCV), alcoholic cirrhosis, aflatoxin-contaminated foods, and excess body weight [18,19]. Due to early detection and a systemic therapy of surgery combined with adjuvant chemotherapy, targeted treatment, or immunotherapy, the mortality rate of HCC has declined in the last three decades [4]. However, the 5-year survival rate of patients with advanced HCC is still low, which is mainly due to tumor advances [20]. Therefore, it is important to understand the molecular mechanisms underlying HCC to identify an effective target for prevention and treatment. Recent studies have focused on the relationships between HCC, TMBs, and immunity and have confirmed that HCC with a high tumor mutation burden (TMB-H) may generate immunogenic neoantigens. The increased production of neoantigens is positively related to the infiltration of immune cells, especially for the count of macrophages and CD4^+^ and central memory T cells [21,22]. The infiltration changes of immune cells are the basis for a good response to immunotherapy [23]. However, none of these have been applied clinically; therefore, we used bioinformatics tools to analyze HCC-associated TMBs and to identify potential immune biomarkers for the diagnosis and prognosis of HCC.

In the present study, we performed a comprehensive biological analysis on the relationship between tumor somatic mutational profiles and immunity for HCC. To understand the functions and associations of these TMB-associated DEGs, GO analyses were performed. The result showed that DEGs are mainly enriched in nucleocytoplasmic and nuclear transport, and previous studies have confirmed that nucleocytoplasmic and nuclear transport are closely associated with the development of tumorigenesis [24,25]. Further studies have confirmed that nucleocytoplasmic and nuclear transport are closely related to HCC metastasis [26]. These studies suggested that the DEGs of TMBs may be closely correlated with the metastasis of HCC. By constructing a PPI network, we found that *USP39*, *RBM22*, *SNRPD1*, *CPSF3*, *SRSF1*, *SRSF3*, *HSPA8*, *HNRNPU*, *SRSF4*, *CWC27*, *EFTUD2*, *ALYREF*, *NCBP2*, *SNRPA1*, and *POLR2D* may play pivotal roles in the development of HCC. There was no research to investigate the relationship between HCC and the genes of *RBM22*, *SRSF4*, *CWC27*, and *POLR2D*, which would provide us a new research direction. In addition, we further used GO annotation semantics to investigate the functional similarity of key DEGs, and a strong biological functional similarity was found between *SRSF1*, *SNRPA1*, *SRSF3*, *SRSF4*, and *ALYREF*. *SNRPA1* was reported to promote HCC proliferation through activating the mTOR-signaling pathway [27], and the phosphorylation of *SRSF3* by *PPM1G* could result in the proliferation, invasion, and metastasis of HCC [28]; furthermore, *ALYREF* was significantly correlated to both advanced tumor-node-metastasis stages and poor HCC prognosis [29], which is similar to our results. However, we have not found any reports focused on the effects of *NCBP2* in HCC, which may have helped us to find new immunotherapy targets in HCC; however, it is worth considering for further investigation in future studies. In addition, the pathway enriched by GSEA mainly involved fatty acid metabolism, hypoxia, and the P53 pathway. Fatty acid metabolism was reported to be correlated with the advance of HCC and simultaneously influenced the infiltration of immune cells [30]. Both hypoxia and the mutation of P53 were also reported to lead to the metastasis of HCC [31,32]. The above studies are similar to our results, suggesting that the conclusions of the present study are accurate.

*NCBP2*, also known as *CBP20* or *NIP1*, can bind to the monomethylated 5′ cap of nascent pre-mRNA. *NCBP2* has an RNP domain usually found in RNA-binding proteins and contains the cap-binding activity [33,34]. It has been reported that *NCBP2* regulates proliferation, metastasis, and apoptosis in multiple cancers [35,36], and accumulating evidence suggests that *NCBP2* may serve as a biomarker for carcinogenesis and cancer progression. For example, NCBP2 was upregulated in an acute lymphoblastic leukemia rearrangement child patient (r ALL) compared with non-r ALL patients. Childhood ALL patients with high expressions of NCBP2 had significantly poorer overall survival rates [37]. The latest study revealed that NCBP2 was overexpressed in the high-risk group of acute myeloid leukemia (AML) and was negatively correlated with survival [38]. In the present study, the results showed that NCBP2 was upregulated in multiple cancers and played a detrimental role at the LIHC stage, and NCBP2 expression was significantly related to another five cancers, including KICH, KIRP, LUAD, PAAD, and PRAD, and was negatively correlated with the over survival of those cancers. Moreover, the present study revealed that the expression of NCBP2 was significantly upregulated in HCC compared with adjacent liver tissues according to the Human Protein Atlas database, and NCBP2 played a detrimental role in the OS of HCC patients. The antisense gene protein NCBP2-AS2 (transcribed from the antisense DNA strand of the gene NCBP2) also plays an important role in multiple tumors. A study has revealed that NCBP2-AS2 was overexpressed in hypoxic-cancer-associated fibroblasts, and it can promote the secretion of pro-angiogenic factor VEGFA, consequently reducing VEGF/VEGFR downstream signaling, which leads to tumor metastasis and reduces the efficacy of therapy [39]. Furthermore, LncRNA NCBP2-AS2 was upregulated in lung squamous cell carcinoma samples compared with lung adenocarcinoma samples and adjacent tissues and promoted cell proliferation and metastasis, as well as the invasive and inhibited apoptosis of SCC cells via the TAp63/ZEB1-regulating pathway [40]. LncRNA NCBP2-AS2 also could promote HCC cell growth and proliferation through regulating KRASIM [41]. In conclusion, NCBP2 is overexpressed in multiple cancers compared with adjacent normal tissues, and high expressions of NCBP2 were significantly correlated with poor OS in HCC. However, further research is needed to establish diagnostic accuracy and treatment with NCBP2 in liver cancer.

To further investigate the role of immune cell infiltration in HCC, TIMER database analysis revealed that the NCBP2 expression was most positively correlated with macrophages (r = 0.551, *p* = 1.97 × 10^−28^) and CD4^+^ T cells (r = 0.484, *p* = 1.37 × 10^−21^). Studies have demonstrated that by infiltrating tumor-associated macrophages (TAMs) at a high level in HCC, target TAM infiltration results in tumor growth inhibition in a mouse HCC model [42,43]. Higher infiltrating fractions of activated memory CD4^+^ T cells were also found in high-risk groups of HCC patients [44,45]. These results showed that the expression level of NCBP2 may be associated with the immune response to the tumor microenvironment of HCC, especially with CD4^+^ T cells and macrophages. In addition, our study investigates the details of 22 types of immune cell infiltrations in HCC, and the results showed that T cells were closely related to follicular helper T cells (TFH), whereas regulatory cells (TReg) showed the weakest interactions with plasmacytoid dendritic cells (pDC), which provided ideas for further investigations regarding the regulation mechanisms of HCC in immune cells, for which no research currently exists. The degree of central memory T cell (Tcm) infiltration was higher in the high-mutation-frequency TP53 samples. Accumulating research has demonstrated that the infiltration of Tcm may help to discover novel treatments for more effective cancer immunotherapies [46,47]. Tcm are functionally and phenotypically distinct monitoring points in the liver, capable of long-lived retention, and well positioned for rapid and potent front-line immunosurveillance [48]. The above studies, combined with our research, have shown that immune cells, especially CD4^+^ T cells, macrophages, and central memory T cells, play important roles in HCC and should be the focus of further studies.

In summary, comprehensive bioinformatic analyses were performed to analyze the predictive value of TMB in HCC prognosis and identified that the expression of NCBP2 was strongly correlated to HCC prognosis. Moreover, immune cell infiltration investigations also suggested that immune cells, especially CD4^+^ T cells, macrophages, and central memory T cells, play important roles in HCC. It is noteworthy that the systematic analysis of TMB-status hub genes in the present study will facilitate an understanding of the role played by TMBs in HCC and contribute to accurate immunotherapeutic treatment. Our findings may serve as a potential guide for targeted immunotherapy and provide ideas for the further development of new immunotherapies. Notwithstanding, more clinical studies and experimental research are needed to verify our findings and explore the molecular mechanisms of TMBs in HCC.

## Figures and Tables

**Table 1:** Univariate and multivariate Cox regressions on clinicopathological characteristics and NCBP2 expression signature.

Variables	Univariate Cox		Multivariate Cox	
	HR (95% CI)	*p* Value	HR (95% CI)	*p* Value
stage2	1.576	0.345	1.367	0.215
stage3	2.205	0.001 **	2.205	0.001 **
stage4	4.575	0.005 *	4.575	0.015 *
Gender male	0.907	0.789	0.907	0.632
B_cell	0.008	0.235	0.008	0.182
CD8^+^ _ T cell	0.005	0.045 *	0.005	0.037 *
CD4^+^ _ T cell	0.014	0.34	0.014	0.22
NCBP2	1.467	0.006 **	1.437	0.046 *

* *p* < 0.05; ** *p* < 0.01; CI: Confidence Interval; HR Hazard Ratio.

## Data Availability

TCGA-LIHC dataset is available at The Cancer Genome Atlas (https://cancergenome.nih.gov/).
